# Formulation Optimization of Hydrodynamically Balanced Oral Controlled Release Bioadhesive Tablets of Tramadol Hydrochloride

**DOI:** 10.3797/scipharm.1001-04

**Published:** 2010-04-18

**Authors:** Bhupinder Singh, Ashu Rani, Babita, Naveen Ahuja, Rishi Kapil

**Affiliations:** University Institute of Pharmaceutical Sciences, UGC Center of Advanced Studies, Panjab University 160 014, Chandigarh, India.

**Keywords:** Drug delivery, Gastroretentive, Mucoadhesive, Experimental design, Floatation

## Abstract

The directly compressible floating-bioadhesive tablets of tramadol were formulated using varying amounts Carbopol 971P (CP) and hydroxy-propylmethyl cellulose (HPMC), along with other requisite excipients. *In vitro* drug release profile, floatational characteristics and *ex vivo* bioadhesive strength using texture analyzer were determined, and systematically optimized using a 3^2^ central composite design (CCD). The studies indicated successful formulation of gastroretentive compressed matrices with excellent controlled release, mucoadhesion and hydrodynamic balance. Comparison of the dissolution profiles of the optimized formulation, with optimal composition of CP:HPMC :: 80.0:125.0, with that of the marketed controlled release formulation other indicated analogy of drug release performance with each other. Validation of optimization study using eight confirmatory experimental runs indicated very high degree of prognostic ability of CCD with mean ± SEM of −0.06% ± 0.37. Further, the study successfully unravels the effect of the polymers on the selected response variables.

## Introduction

Amongst various routes to deliver drugs, oral intake has unambiguously been the most sought after by the patients and manufacturers alike. Using the conventional oral dosage forms several drugs have to be administered quite frequently (i.e., 2–4 times-a-day) resulting, therefore, in high fluctuation in plasma drug levels causing saw-tooth kinetics. Controlled release (CR) systems are designed primarily for reducing the frequency of administration by regulating the drug concentration in the target tissue, ensuring patient compliance and consequently improving the efficacy of drugs [1, 2]. However, the development of a CR drug delivery system (DDS) is precluded by its inability to restrain and localize it within the desired region of gastrointestinal (GI) tract, and the highly variable nature of gastric emptying process [3].

The DDS can improve the controlled delivery of the drugs exhibiting an absorption window by continuously releasing the drug for a prolonged period before it reaches the absorption site, thus ensuring its optimal bioavailability [4, 5]. Various approaches including floating systems, bioadhesive systems, swelling and expanding systems and high density systems have been successfully employed to improve the gastric residence time of DDS [6, 7]. Though highly efficient for gastroretention, the floating systems suffer from a major disadvantage that they are effective only when the fluid level in the stomach is sufficiently high. However, as the stomach empties and the tablet is at the pylorus, the buoyancy of the dosage form may be impeded. This serious limitation can be overcome by making the floating system eventually adhere to the mucous lining of the stomach wall [8]. Floating and bioadhesive DDS, thus, offer the advantages of increased gastric residence, leading to improved bioavailability of drugs esp. with narrow absorption window [8, 9].

Tramadol is a synthetic codeine analogue and weak μ-opoid receptor agonist having an immense potential in analgesia. A specific absorption window limited only to the upper part of the small intestine coupled with high frequency of drug administration (4–6 hourly), small dose (50–100 mg) and short biological half-life (6–8 h) rationally call for the development of its gastroretentive CR product. Highly soluble and permeable, this drug can be safely regarded as a BCS class I therapeutic agent.

Modern optimization techniques using experimental designs are a vital aid to the formulator, as they help in developing the best possible formulation under a given set of conditions, thus saving considerable time, money and developmental effort [10, 11]. Also these systematic techniques are known to provide a depth of understanding and ability to explore and defend the ranges for varied formulation and processing factors. Central composite design (CCD), in this regard, has been frequently employed for the optimization of gastroretentive systems [12, 13]. Hence, the present investigation aims at developing oral CR floating- bioadhesive matrices of tramadol hydrochloride, optimized using a CCD.

## Materials

Tramadol HCl was provided *ex gratis* by M/s Alkem Laboratories Ltd., Mumbai, India and Sun Pharma Ltd., Mumbai, India. Methocel (Hydroxypropylmethyl cellulose, HPMC K100LV) and Carbopol 971P (CP) were obtained as the gift samples form M/s Panacea Biotec Ltd., New Delhi, India and M/s Noveon Pharmaceuticals, USA, respectively. Avicel PH 101 (Microcrystalline cellulose, MCC) and magnesium stearate (MST) were obtained from M/s Signet Chemical Corporation, Mumbai, India, and M/s Loba Chemie Ltd., Mumbai, India, respectively. Porcine gastric mucosa for determining bioadhesive strength was obtained from a local slaughter house of Chandigarh, India. All other chemicals and reagents used were of analytical grade, and were used as received.

## Methods

### Screening of polymers and their levels

During preliminary studies, six polymers *viz*. CP 934P, CP 971P, HPMC K15M, HPMC K100LV, NaCMC and Xanthan gum were investigated for formulating oral CR floating-mucoadhesive matrices of tramadol hydrochloride. Tablets were prepared using each of these polymers, with the ratio of tramadol to polymer kept as 1:2 to 1:4.

Later on, depending upon the results obtained, the polymer blend containing the two polymers *viz.* CP 971P and HPMC K100LV was selected for further investigation. Besides these polymers, the other constituent employed in variable amounts was MCC. To evaluate the inertness of MCC on drug release of tramadol, the drug release of formulation without MCC was compared with formulation containing the maximum amount of MCC employed. The initial study to screen suitable polymer combinations of each blend was carried out using the formula shown in Table 1.

### Formulation of tablets as per the experimental design

Different tablet formulations of tramadol HCl were formulated using varying amounts of the polymers (i.e., CP and HPMC), MST as glidant and lubricant, and MCC as an inert diluent. Table 2 enlists the various compositions employed during the study. Prior to use, tramadol and the polymers, *viz.* CP and HPMC were screened through # 80 mesh sieve (size: 180 μm), while MCC and MS were screened through # 120 mesh sieve (size: 125 μm). All the materials were accurately weighed and mixed intimately in a polythene bag for 10 minutes. The blended mix was subsequently compressed into 440 mg tablets using flat-faced round punches (12.8 mm diameter) fitted to a single-punch tablet compression machine (M/s Cadmach, Ahmedabad, India).

## Experimental Design

A central composite design (CCD) for two factors at three levels each (with α=1), equivalent to a 3^2^ factorial design [14], was selected to optimize varied response variables. The two factors *viz.* CP (i.e., polymer X_1_) and HPMC (i.e., polymer X_2_) were varied in the polymer blends, as required by the experimental design, and the factor levels suitably coded (Table 3). The amount of MS was kept as constant at 5 mg, while MCC was employed as a diluent in a sufficient quantity to maintain a constant tablet weight of 440 mg. Amount of drug released in 16 h (rel_16h_), time taken to release 75% of drug (t_75%_), bioadhesive strength (ρ) and buoyancy time (T_b_) were taken as the response variables.

### Tablet Assay and Physical Evaluation

Ten tablets were pulverized. A quantity of powder equivalent to 20 mg of tramadol was shaken with 60 ml of methanol for 10 min. The resulting suspension was heated to 60 °C and shaken for 15 minutes. The contents were cooled and diluted to 100 ml with methanol and filtered (Whatman^®^ grade 597 filter paper, M/s Whatman International Ltd., Kent, UK). Spectrophotometric absorbance of the filtrate was measured at a λ_max_ of 273 nm using a double beam UV/VIS spectrophotometer (Geaesys 6, M/s Thermo Fisher Scientific, Waltham**,** USA). The content of tramadol was determined using a previously constructed standard calibration plot, taking molar extinction coefficient as 2098.6.

Tablets were also evaluated for hardness using an electronic hardness tester (EH-01P, M/s Electrolab Instruments, Mumbai, India, n = 6), friability using Roche friabilator (M/s Tropical Lab Equipment, Mumbai, India, n = 6), weight variation using Analytical balance (AE 240, M/s Mettler Toledo, Greifensee, Switzerland, n = 10), and thickness using Vernier Callipers (M/s Baker Gauges Ltd., Pune, India, n = 10).

### In Vitro Drug Release Studies

Dissolution studies were carried out on all the tablet formulations in triplicate, employing USP XXX [15] paddle method (Apparatus 2, M/s Pharma Test Apparatebau AG, Hainburg, Germany) at 50 rpm and 37 ± 0.5 °C, using simulated gastric fluid (SGF) pH 1.2 without pepsin as the dissolution medium. An aliquot of sample was withdrawn periodically at suitable time intervals and volume replaced with an equivalent volume of plain dissolution medium. Samples were analyzed spectrophotometrically at 271 nm. Drug release data obtained during *in vitro* dissolution studies were analyzed using ZOREL software [16] with in-built provisions for applying the correction factor for volume and drug losses during sampling [17]. Drug release data were fitted into Korsemeyer-Peppas model for swollen matrices [18, 19], as described by Eq. 1.

Eq. 1. $$\frac{M_{t}}{M_{\infty }}=k_{1}\cdot t^{n}+k_{2}\cdot t^{2n}$$

where, ***M_t_*** is amount of drug released at time ‘t’, ***M_∞_*** is amount of drug released at an infinite time, ***k_1_*** is the magnitudinal contribution of diffusion mechanism, ***k_2_*** is the magnitudinal contribution of polymer relaxation mechanism, and n is the Fickian diffusion coefficient. Based on the phenomenological analysis, the type of release, *i.e.*, whether Fickian, non-Fickian (anomalous) or zero-order, was predicted. The value of *t_75%_* was calculated using Stineman interpolation option of the GRAPH 2.0 software (M/s Micromath Inc., Saint Louis, USA).

### Bioadhesion Studies and Duration of Buoyancy

Porcine gastric mucosa was utilized as the model membrane for *ex vivo* bioadhesive strength determination of various formulations. The mucosal membrane was excised by removing the underlying connective tissue and was placed on the base of Texture Profile Analyzer (TAX TEE 32, M/s Stable Microsystems, Surrey, UK). A tablet was attached to the stainless steel probe fixed to the mobile arm of the texture analyzer. The area of contact of mucosa was moistened with 50 μL of SGF. The mobile arm was lowered at a rate of 0.5 mm/s until a contact with the membrane was made. A contact force of 10 g was maintained for 300 s, after which the probe was withdrawn from the membrane. The peak detachment force was recorded as a measure of bioadhesion.

### Determination of Buoyancy Duration

The duration for which the formulation floats in the dissolution medium, in the upper one-third of the dissolution vessel, was determined periodically after every 15 min, by careful visual observation during the dissolution run [20].

### Determination of Specific Gravity

The specific gravity of tablets was determined by displacement method, using benzene as a displacing medium [21]. A plethysmometer was employed to measure tablet density. Firstly, the instrument was calibrated using benzene (density: 0.8723 g/cc) for its volumetric capacity. Benzene was filled up to a mark in the capillary of the instrument. Subsequently, five tablets of known mass were dropped in wider mouth of the plethysmometer. The system was kept undisturbed for 1 min to let benzene displace the air in the pores of the tablets. After that, the displacement in the volume of benzene in the side capillary was noted. Knowing the weight and volume occupied by the tablets, density of five tablets was determined.

### Optimization Data Analysis and Validation of Optimization Model

The response variables which were considered for systematic DoE optimization included t_75%_, rel_16h_, T_b_ and ρ. For the studied design, the MLRA method was applied to fit full second-order polynomial equation with added interaction terms to correlate the studied responses with the examined variables using Design expert ver. 6.0.10 software (Stat-Ease, Minneapolis, USA). The polynomial regression results were demonstrated for the studied responses. Finally, the prognosis of optimum formulation was conducted using a two-stage brute force technique using MS-Excel spreadsheet software. First, a feasible space was located and second, an exhaustive grid search was conducted to predict the possible solutions. The region of optimality was also ratified using overlay plots, drawn using the Design Expert^®^ software. Eight formulations were selected as the confirmatory check-points to validate RSM [5, 20, 22]. The observed and predicted responses were critically compared. Linear correlation plots were constructed for the chosen eight optimized formulations (CP:HPMC :: 80:125, 92.8:129, 140.8:149.75, 137.6:134, 123:166, 118.4:169, 112:175, 126.4:175). The residual graphs between predicted and observed responses were also constructed separately, and the percent bias (= prediction error) was calculated with respect to the observed responses.

### Comparison of Drug Release with Marketed Formulation

Drug release profile of the optimized formulations was compared with two marketed brands of once-a-day formulations, Tramazac™ TC and Dolfre™ SR, each containing 100 mg of tramadol hydrochloride per tablet.

## Results and Discussion

### Selection of Polymers and Their Levels

Six polymers *viz*. two grades of CP (i.e., CP 934P and CP 971P), HPMC (i.e., K15M and K100LV), sodium CMC and xanthan gum were selected for the preliminary studies, owing to their reported potential of release rate controlling ability, bioadhesive strength, non-toxicity, non-irritancy, stability at GI pH and compatibility with drug. [23–25].

Dissolution parameters of all the six selected polymers were studied by formulating them into tablet dosage forms containing varying drug: polymer ratios ranging between 1:2 and 1:4. Out of all the polymers, CP 971P was found to be the most promising in regulating the drug release profile, followed by xanthan gum, sodium CMC, CP 934P, HPMC K15M and HPMC K100LV, as revealed by the high values of t_70%_ associated with them. The high potential of CP in controlling drug release and imparting bioadhesive characteristics to the system has already been proved with fruition in our laboratories too with mucoadhesive tablets of atenolol [12], diltiazem HCl [26] and hydralazine HCl [5]. Further, the compressed matrices formulated with HPMC K100LV, HPMC K15M and sodium CMC were found to be buoyant at all the studied levels with the order of floating time as: HPMV K100LV > HPMCK15M >> sodium CMC. The results are in consonance with earlier literature findings reporting high floatation potential of these cellulosic polymers [27].

The lowest percentage of the hydrophobic substituents (methoxyl group) and the highest amount of hydrophilic substitution (hydropropoxyl) impart Methocel K series with the fastest rate of hydration, as compared to the E and F series. Also, it is conceivable that for highly soluble drugs, an inadequate polymer hydration rate may lead to significant dose dumping due to quick penetration of fluids into tablet core. Hence, amongst the various substitutions types, the rapidly hydrating HPMC 2208 (Methocel K) is considered ideal for regulating the release of tramadol hydrochloride. Higher viscosity grades like Methocel K15 and K15CR were deemed unsuitable, as these usually yield drug blood levels in sub-therapeutic range. Moreover, literature documents that the low-viscosity grades (e.g., HPMC K100LV) were found to be more beneficial than high-viscosity ones (e.g., HPMC K4M, HPMC K15M) in improving the floating properties [27]. For further product development studies, therefore, HPMC K100LV rather than HPMC K15M was chosen.

The successful use of the polymer combination of CP and HPMC has already been documented in various literature reports in attaining excellent CR characteristics [5, 8, 12, 28]. Further, a combination of ionic polymer (like CP) and nonionic polymer (like HPMC) is known to provide the formulation with controlled drug release and/or desired mucoadhesive properties [26, 29].

For preliminary batch of tablets, drug release, as discerned from t_75%_ values, was found to be better extended with increase in levels of either polymer. However, rel_16h_ was found to be less than 89% in all the cases. Hence, it was planned to investigate levels of factors (80–160 mg for CP and 125–175 mg for HPMC), different from the ranges studied during pre-optimization studies (100–150 mg for CP and 150–200 mg for HPMC). Buoyancy time was found to decrease with increase in CP content, while reverse was the trend with increasing HPMC content.

### Selection of Other Excipients

Water insoluble and water immobile excipients like MCC and dibasic calcium phosphate have been employed with fruition as inert diluents while achieving CR [20]. In the present study, tablets prepared using dibasic calcium phosphate sank immediately to the bottom of the dissolution beaker ostensibly due to its high density (0.780g/cc). Therefore, MCC with lower density (0.337g/cc) was employed as a diluent in the current studies. Further during the preliminary studies, the drug release profiles of the formulations with maximum amount of MCC employed and without MCC were found almost to superimpose over each other (f_1_ = 84.9).

Selection of concentration of MST as ∼1% was based on earlier studies carried out in our laboratories as it was found to be the adequate concentration to attain good powder flow characteristics and die ejection [5, 12]. The same was ratified in our preliminary experimental studies with tramadol tablets, too.

### Drug Content and Physical Evaluation

The assayed content of drug in various formulations varied between 98.9% and 100.5% w/w with mean ± SD as 99.7 ± 0.5%. Tablet weights varied between 439.1 and 442.4 mg (440.8 ± 2.2 mg), and thickness between 3.2 and 3.4 mm (3.3 ± 0.1 mm). Tablets require a certain amount of strength or hardness, and resistance to friability, to withstand the mechanical shocks of handling during their manufacture, shipping and packaging. The hardness of a tablet is closely related to its disintegration time and dissolution, and eventually its drug release rate [30]. Tablet hardness monitoring, therefore, is especially important for drug products which possess real or potential bioavailability problems or those sensitive to altered dissolution release profiles as a function of the compressive force applied. Representative tablets tested from each batch possessed hardness values hardness values ranging between 52.98 N and 70.24 N (60.21 ± 4.2 N), indicative of adequate strength to provide good tablet disintegration and dissolution profiles and to prevent friability losses. All the tablets tested from each batch exhibited friability values ranging between 0.37% and 0.65% w/w (0.50 ± 0.14%), far less than the limit of 1% w/w, generally considered as acceptable by the official compendia [31, 32]. Marginal variation in tablet hardness and friability could be attributed only to the random causes, but not to the matrix composition. This absence of any significant inter- and intra-batch variability in tablet hardness, friability and thickness, ruled out any plausibility of any change in compression pressure, and consequently in drug dissolution.

### In Vitro Drug Release Studies

Table 4 enlists various dissolution parameters computed for all the CR bioadhesive formulations. Summary of the dissolution parameters, indicated in Table 3, shows that the value of ***n*** varies between 0.4502 and 0.5719, delineating non-Fickian release behavior. The values of ***n*** show increasing trend with increase in HPMC content, even at higher CP levels. However at low levels of CP 971P, ***n*** seems to bear a nonlinear relationship with HPMC. The value of ***n*** decreases as HPMC increases from low to intermediate levels, but enhances with further increase in HPMC to high levels. The table also shows a rising trend in the values of ***n*** as the content of CP is increased with significant increase at the highest levels of CP. Overall, the current results seem to be in agreement with the previous findings indicating ambiguous relationship of n with change in polymer composition [12, 26]. As depicted in the table, the values of k followed a declining trend with increase in the amount of either polymer.

Relatively much higher magnitude of ***k_1_*** vis-à-vis ***k_2_*** clearly show that the drug release was predominantly Fickian diffusion, with the contribution of polymer relaxation as nearly negligible. This is in consonance with the earlier findings that a mixture of HPMC with CP resulted in the reduction of polymer viscosity due to reduced hydration [5, 26, 33]. This reduction of viscosity could facilitate drug diffusion through polymer hydrogel. Table 3 reveals that the overall rate of drug release tended to decrease with increase in concentration of HPMC or CP. Similarly, the values of Rel_16h_ decreased drastically with increase in the polymer content. As much as 18% of drug is retained in the matrix till 16 hours when the highest levels of both the polymers were employed. Plausibly, it can lead to appreciable diminution in the extent of drug absorption. Conversely, the values of t_75%_ were found to enhance markedly from 7.12 h to 11.99 h from low to high levels of both the polymers.

### Bioadhesive Strength Determination

**Fig. 1.**
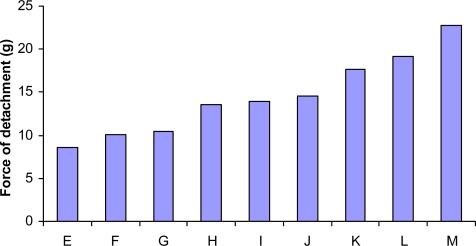
Bioadhesive strengths of the formulations prepared as per experimental design

A distinct increase in the bioadhesive strength is observed with an increase in the amount of either polymer (CP or HPMC), which is in agreement with literature [12, 26, 34, 35]. Hydrogels swell rapidly in contact with hydrated mucous membrane, resulting in reduced glass transition temperature and increased uncoiling along with an increased mobility of polymer chains [12]. This tends to increase the adhesive surface for maximum contact with mucin and flexibility for interpenetration with mucin. Although the maximum value of bioadhesive strength was attained at the highest levels of both the polymers, yet the effect of CP was found to be more pronounced than that of HPMC. The bar diagram for detachment force (Fig. 1) pictographically depicts the change in bioadhesive strength of tablets with a change in the polymer level(s).

### Buoyancy Time and Specific Gravity

**Fig. 2.**
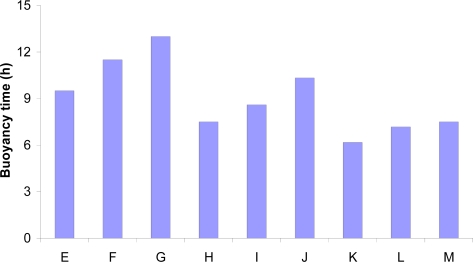
Buoyancy time of the formulations prepared as per experimental design

It is a well-documented fact that swelling is a vital factor to ensure floatation [36–38]. To obtain adequate floating, the balance between swelling and water acceptance must be restored [20]. Buoyancy time (T_b_) of the tablets increased in a linear fashion with increase in HPMC content, owing ostensibly to swelling (i.e., hydration) of the hydrocolloid particles on the tablet surface, resulting ultimately in an increase in the bulk volume. The air entrapped in the swollen polymer maintains a density less than unity and confers buoyant character to these dosage forms. With increase in CP content, however, buoyancy time decreases in a linear trend, probably due to higher density of CP (1.76 g/cc) than that of HPMC (1.28 g/cc). The bar diagram for buoyancy time (Fig. 2) corroborates the significant positive and negative influence of HPMC and CP on floatation, respectively. But, it is of interest to mention that the presence of CP could possibly aid in retaining the tablet following oral ingestion within the stomach by assisting in the adhesion of the dosage form on the gastric wall, which in turn, may aid in enhancing the tablet gastric retention time [28]. Tablet density of all the formulations was found to be lower than the density of gastric contents (1.004 g/cc), which satisfies the major criterion for a dosage form to float [39, 40]. Rather on water absorption into the polymer matrix and subsequent swelling, the density of swollen but intact tablet is further going to reduce in magnitude.

High degree of correlation was found between buoyancy time (T_b_) and tablet density for all the formulations (r^2^ = 0.9901). As tablet density decreases, buoyancy time increases in a linear fashion. This construes that floating tendency of the matrix formulation is an inverse function of its density. Hence, to estimate the effect of formulation factors on the floatation characteristics, buoyancy time (but not density), was taken as the response parameter. Analogously, MDT and t_75%_ were also found to be highly correlated with each other (r^2^=0.9739). And on the similar heels, t_75%_ (and not MDT) was taken as response parameter to declare extended drug release

### Exploration of Polymer Mechanism using RSM

Quite high values of *R*^2^ of the MLRA coefficients for all four responses, ranging between 0.9853 and 1.0000, vouch high prognostic ability of the RSM polynomials. Seven coefficients (β_1_ to β_7_) were calculated with β_0_ representing the intercept, and β_3_ to β_7_, representing the various quadratic and interaction terms (Eq. 2).

Eq. 2. $$Y=\beta_{0}+\beta_{1}X_{1}+\beta_{2}X_{2}+\beta_{3}X_{1}X_{2}+\beta_{4}X_{1}^{2}+\beta_{5}X_{2}^{2}+\beta_{6}X_{1}X_{2}^{2}+\beta_{7}X_{2}X_{1}^{2}$$

**Fig. 3.**
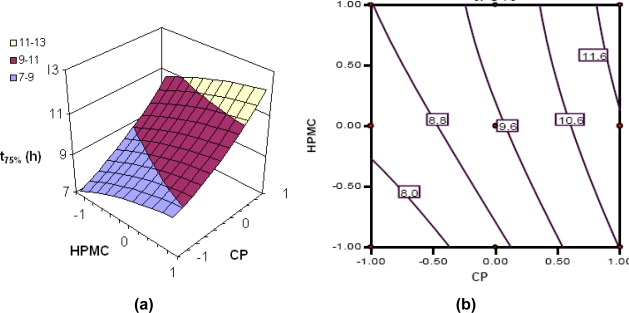
(a) Response surface plot showing the influence of CP and HPMC on the value of t_75%_ of floating-bioadhesive tablet formulations of tramadol; (b) the corresponding contour plot

**Fig. 4.**
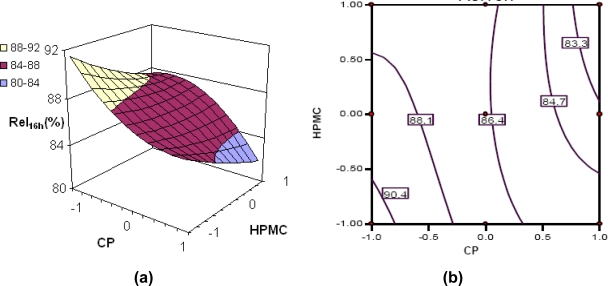
(a) Response surface plot showing the influence of CP and HPMC on the value of rel_16h_ of floating-bioadhesive tablet formulations of tramadol; (b) the corresponding contour plot

**Fig. 5.**
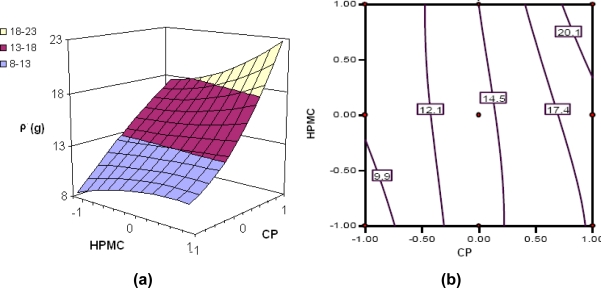
(a) Response surface plot showing the influence of CP and HPMC on the value of ρ of floating-bioadhesive tablet formulations of tramadol; (b) the corresponding contour plot

**Fig. 6.**
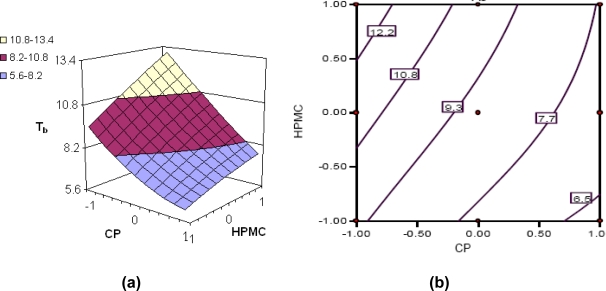
(a) Response surface plot showing the influence of CP and HPMC on the value of T_b_ of floating-bioadhesive tablet formulations of tramadol; (b) the corresponding contour plot

Various response surfaces and contour plots are depicted in Fig. 3 to 6. Fig. 3a to 6a portray the 3-D response surface plots, while Fig. 3b to 6b are the corresponding 2-D contour plots for the studied response variables. Fig. 3a depicts quite linear increasing trend in the values of t_75%_ with augmentation of CP, and nearly linear increasing trend with HPMC fractions. Nevertheless, the influence of CP is distinctly far more significant than that of HPMC, indicating that the former has better release sustaining properties for tramadol than the latter. The same is being confirmed from the corresponding contour plot (Fig. 3b) showing declining linear contour lines. Hence, the higher levels of CP have to be complemented with lower levels of HPMC and vice-versa to maintain the value of t_75%_ at a constant level.

Fig. 4a and 4b reveal a decline in the value of Rel_16h_ with an increase in the concentration of each of the polymers, i.e., CP and HPMC, the influence of CP being much more pronounced. At low levels of HPMC, a distinct linear decreasing trend is followed with increase in CP levels.

Nonlinear descending contour lines in Fig. 4b further elucidate that the variation in Rel_16h_ is an enigmatic function of the polymer levels, the effect of HPMC being less significant.

Fig. 5a shows nearly linear ascending patterns for the values of bioadhesive strength as the content of either polymer is increased, once again, the effect, being more prominent with CP than with HPMC. At high levels of the polymers, however, the response surface takes a slight curvilinear shape. Maximum bioadhesive strength was observable at the highest levels of both the polymers *viz.* CP and HPMC. The corresponding contour plot (Fig. 5b) also reveals nearly decreasing trend at all the factor levels. Nearly vertical contour lines corroborate that only CP influences the ρ values significantly. The results are in consonance with literature reports stating high contribution of carbomers in attainment of bioadhesive strength in hydrophilic matrices [5, 12, 28].

The response surface (Fig. 6a) vividly connotes that HPMC is contributing significantly towards attaining floating characteristics to the formulation. The higher levels of CP, on the other hand, were counter-productive in imparting the buoyant character to the drug delivery devices, as nearly linear decreasing trend in T_b_ is clearly discernible with increased CP levels. Further, the 3D plot also reveals that the positive influence of HPMC in achieving higher values of T_b_ is relatively less pronounced than the negative influence of CP on the same. The corresponding 2-D contour plot (Fig. 6b) also depicts a curvilinear ascending pattern for the values of buoyancy time (T_b_) as HPMC content increases. Maximum value of buoyancy time is discernible at the highest levels of HPMC and the lowest levels of CP, while the converse is also true to attain the minimum.

### DoE Validation and Selection of Optimum Formulation

**Fig. 7.**
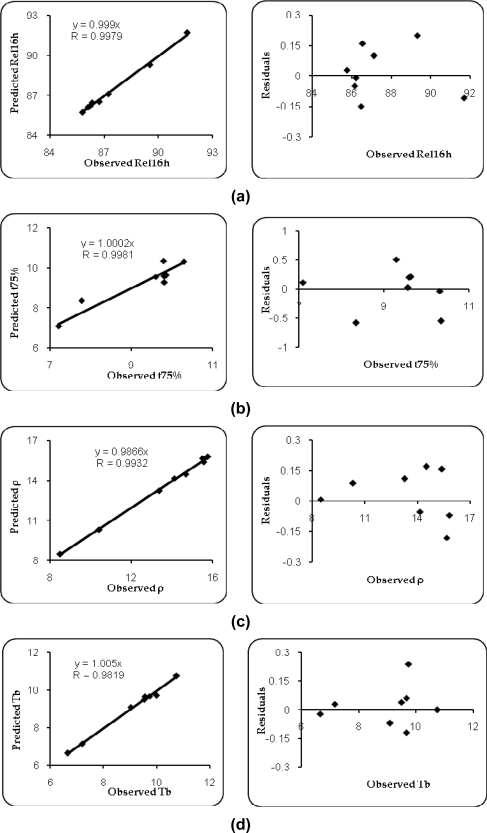
Linear and residual plots between observed and predicted values of (a) Rel_16h_, (b) t_75%_, (c) ρ, (d) T_b_

Upon comparison of the observed responses with those of the anticipated ones, the prediction error varied between −6.9 and 5.4 % with overall mean ± SD as −0.06 ± 0.37%. Linear correlation plots (Fig. 7) drawn between the predicted and observed responses after forcing the line through the origin, also demonstrated high values of r (0.9819 to 0.9981), indicating excellent goodness of fit in each case (*p* < 0.001). The corresponding residual plots show nearly uniform and random scatter around the mean values of response variables.

The optimum formulation was selected by “trading off” various response variables and adopting the following maximizing criteria: t_75%_ ≥ 7.1 h; rel_16h_ > 89%; ρ > 8.0 g and T_b_ > 8.5 h. Upon comprehensive evaluation of grid searches, the formulation (CP: 80.0 mg and HPMC: 125.0 mg) fulfilled the optimal criteria of best regulation of the release rate, floating and bioadhesive characteristics with t_75%_ of 7.10 h, rel_16h_ of 91.71%, ρ of 8.5 g and T_b_ of 9.68 h. Thus, besides controlling drug release, the formulation has definite gastroretentive potential to retain the drug in the gastric environment and upper part of intestine.

### Comparison of Release Performance with Marketed Brands

Table 5 shows the comparison of dissolution parameters of the marketed brands with the optimized formulation. Complete drug release was observed at 24 h in all the three studied formulations.

**Fig. 8:**
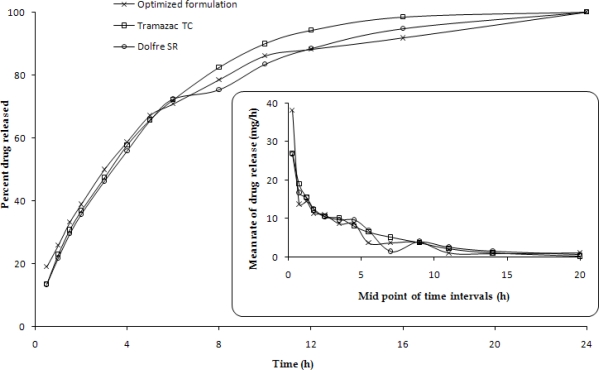
*In vitro* drug release profiles of the optimized formulation and the two marketed formulations. The inset shows the corresponding rates of drug release.

Drug release from the optimized formulation at 12 h (88.15%) was found to be closer to that of Dolfre™ SR (88.41%). Similarly, the release parameters like t_70%_, Rel_16h,_ MDT, K, ***n*** were quite close to each other. Further, the values of similarity factor, f_1_, at periodic intervals of 8 h of both the marketed formulations vis-à-vis the optimised formulation, ranged between 70.16 and 75.49, unambiguously corroborating the sameness of the release profiles. Fig. 8 portrays the respective release profiles of the marketed formulations (esp. Dolfre™ SR) and optimized formulation superimposed over each other also indicating almost analogy of release performance with each other. Thus, the studies conclude successful development of gastroretentive CR formulation of tramadol capable of maintaining similar drug release profiles as observed with the marketed CR products and delivering the drug at its preferred site of absorption in the GI tract.

## Conclusions

The fluctuation in plasma levels of the drug and low patient compliance due to this high frequency of administration can be overcome only by formulating it as a CR once-daily DDS. Accordingly, the present studies aimed at formulating tramadol into a gastroretentive floating-bioadhesive system, preferred due to its ability of retaining the DDS in GIT and improving bioavailability esp. for drugs exhibiting specific absorption window in GI tract. But it was a Herculean task to attain the required floatational properties and bioadhesive potential in the formulation using blends of polymers like carbomers and methylcelluloses because of the diverse nature of these polymers. Carbomers, though, are very bioadhesive but being heavier in density, are considered unsuitable to impart buoyant characteristics to the formulation. On the other hand, the lighter hydrophilic methylcelluloses impart floatation but are less effective as bioadhesives. Only systematic studies using DoE optimization could surmount this hiccup of balancing optimal floatation with bioadhesion using this polymer combination. The choice of experimental design, i.e., a 2-factor CCD, was found to be highly appropriate, as it can detect any non-linearity in factor-response relationship with minimal expenditure of developmental effort and time. The optimized formulation exhibited excellent CR, bioadhesive and floatational characteristics vouching the success of the experimental approaches followed. Besides identical drug release profile to that observed with the marketed CR formulations, the optimized formulation exhibited excellent floatational and bioadhesive properties too using a synergistic blend of effective and cost-effective polymers. Hence, the studies can be safely regarded as a platform technology in the manufacture of gastroretentive CR formulations of BCS class I drugs as generic products, where matching of the drug release profiles with that of the innovators’ is the major criterion.

## Figures and Tables

**Tab. 1.** General composition of tramadol hydrochloride matrices during initial studies

**Ingredient**	**Amount (mg)**
Tramadol hydrochloride	100
CP 971P	100–150
HPMC K100LV	150–200
Magnesium stearate	5
Microcrystalline cellulose	q.s. to 400

**Tab. 2.** Composition of gastroretentive tramadol tablets

**Ingredient**	**Amount (mg)**
Tramadol hydrochloride	100
Carbopol	80–160
Hydroxypropyl meyhyl cellulose	125–175
Magnesium stearate	5
Microcrystalline cellulose	q.s.

**Tab. 3.** Factor Combinations as per the Chosen Experimental Design

**Formulation code**	**Experimental Trial No.**	**Coded Factor levels**		
		**X_1_**	**X_2_**	
E	1	−1	−1
F	2	−1	0
G	3	−1	1
H	4	0	−1
I	5	0	0
J	6	0	1
K	7	1	−1
L	8	1	0
M	9	1	1

**Translation of coded levels in actual units**			
Coded Level	−1	0	1
X_1_: CP (mg)	80	120	160
X_2_: HPMC (mg)	125	150	175

**Tab. 4.** Overall dissolution parameters for all the floating-bioadhesive tablet formulations of tramadol prepared using different amounts of CP 971P and HPMC K100LV of polymer blend as per central composite design

**Code**	**n**	**K**	**k_1_**	**k_2_**	**rel_16h_ (%)**	**t_75%_ (h)**	**Drug release rate (mg/h) (Mean ± S.D.)**
E	0.4572	0.2844	1.3439	−0.0035	91.76	7.12	9.25±8.93
F	0.4502	0.2772	1.3329	−0.0045	88.75	8.44	8.88±8.76
G	0.4626	0.2653	1.3160	−0.0019	87.76	8.73	8.75±8.65
H	0.4745	0.2555	1.3037	0.0001	86.84	8.80	8.71±8.56
I	0.4842	0.2483	1.2921	0.0028	87.18	9.15	8.62±8.25
J	0.4889	0.2420	1.2828	0.0039	86.41	10.20	8.50±8.07
K	0.5093	0.2298	1.2659	0.0080	86.04	10.58	8.36±7.54
L	0.5366	0.2073	1.2306	0.0141	83.20	11.72	8.00±6.68
M	0.5719	0.1890	1.2056	0.0198	81.91	11.99	7.73±6.06

**Tab. 5.** Comparison of release performance of the optimized formulation with marketed brands of tramadol hydrochloride

**Formulation**	**t_70%_ (h)**	**Rel_12h_ (%)**	**Rel_16h_ (%)**	**MDT (h)**	**n**	**k**	**Similarity Factor**		
							**8h**	**16h**	**24h**
Tramazac™ TC	5.72	94.24	98.50	4.802	0.5407	0.2430	75.49	70.16	70.97
Dolfre™ SR	5.58	88.41	94.82	5.143	0.5378	0.2355	72.03	73.46	74.24
Optimized formulation	5.76	88.15	91.71	4.920	0.4569	0.2848	–	–	–
